# Effects of anti-inflammatory therapies on glycemic control in type 2 diabetes mellitus

**DOI:** 10.3389/fimmu.2023.1125116

**Published:** 2023-03-01

**Authors:** Dandan Li, Jiaxin Zhong, Qirui Zhang, Jingjing Zhang

**Affiliations:** ^1^ National Clinical Research Center for Metabolic Diseases, Metabolic Syndrome Research Center, Key Laboratory of Diabetes Immunology, Ministry of Education, and Department of Metabolism and Endocrinology, The Second Xiangya Hospital of Central South University, Changsha, Hunan, China; ^2^ Department of General Surgery, The Second Xiangya Hospital of Central South University, Changsha, Hunan, China

**Keywords:** type 2 diabetes, anti-inflammatory therapies, antidiabetic drug, clinical trial, meta-analyses

## Abstract

**Background:**

The overall evidence base of anti-inflammatory therapies in patients with type 2 diabetes mellitus (T2DM) has not been systematically evaluated. The purpose of this study was to assess the effects of anti-inflammatory therapies on glycemic control in patients with T2DM.

**Methods:**

PubMed, Embase, Web of Science, and Cochrane Library were searched up to 21 September 2022 for randomized controlled trials (RCTs) with anti-inflammatory therapies targeting the proinflammatory cytokines, cytokine receptors, and inflammation-associated nuclear transcription factors in the pathogenic processes of diabetes, such as interleukin-1β (IL-1β), interleukin-1β receptor (IL-1βR), tumor necrosis factor-α (TNF-α), and nuclear factor-κB (NF-κB). We synthesized data using mean difference (MD) and 95% confidence interval (CI). Heterogeneity between studies was assessed by *I^2^* tests. Sensitivity and subgroup analyses were also conducted.

**Results:**

We included 16 RCTs comprising 3729 subjects in the meta-analyses. Anti-inflammatory therapies can significantly reduce the level of fasting plasma glucose (FPG) (MD = - 10.04; 95% CI: -17.69, - 2.40; P = 0.01), glycated haemoglobin (HbA1c) (MD = - 0.37; 95% CI: - 0.51, - 0.23; P < 0.00001), and C-reactive protein (CRP) (MD = - 1.05; 95% CI: - 1.50, - 0.60; P < 0.00001) compared with control, and therapies targeting IL-1β in combination with TNF-α have better effects on T2DM than targeting IL-1β or TNF-α alone. Subgroup analyses suggested that patients with short duration of T2DM may benefit more from anti-inflammatory therapies.

**Conclusion:**

Our meta-analyses indicate that anti-inflammatory therapies targeting the pathogenic processes of diabetes can significantly reduce the level of FPG, HbA1c, and CRP in patients with T2DM.

## 1 Introduction

Obesity and type 2 diabetes mellitus (T2DM) are associated with decreased physical activity and unhealthy high-calorie diets. Obesity is related to insulin resistance and is a crucial risk factor for the development of T2DM (1). Chronic low-grade inflammation plays an important role in the pathogenesis of diabetes and the development of diabetic complications (2, 3). Inflammation has been seen in the pancreatic islets, liver, muscle, adipose tissue, and the sites of diabetic complications (4). Long-term inflammation that occurs in adipose tissue can lead to systemic inflammation and contribute to insulin resistance. In the presence of insulin resistance, β cells secrete more insulin to maintain normal glucose control. Inflammation impairs β cell function and induces β cell apoptosis, and T2DM happens when insulin production fails to reach the insulin needs (5).

Many proinflammatory cytokines and inflammation-associated nuclear transcription factors are related to impaired insulin secretion and contribute to the pathogenesis of T2DM, including interleukin-1β (IL-1β), tumor necrosis factor-α (TNF-α), and nuclear factor-κB (NF-κB) etc. (4, 6–8). High concentration glucose can induce IL-1β production and secretion from human pancreatic β cells, and IL-1β was observed in β cells in diabetic patients (9). IL-1β is involved in β cell apoptosis and partially dependent on the activation of NF-κB (10). Obesity can activate the NF-κB signaling pathway, which plays an important role in the development of insulin resistance (8). TNF-α is also involved in β cell apoptosis (11), and more TNF-α expression was found in adipose tissue in obese than lean people, and the plasma level of TNF-α was elevated in patients with T2DM (6, 7).

Anti-inflammatory treatments can improve insulin sensitivity and β cell function in patients with insulin resistance or T2DM (12). Treatments of diabetes focused on inflammation can benefit many inflammatory tissues at the same time, which is less likely to induce hypoglycemia (13). Small molecules or antibody-based molecules targeting inflammatory cytokines, cytokine receptors, or inflammation-associated nuclear transcription factors, such as IL-1β, interleukin-1β receptor (IL-1βR), NF-κB, and TNF-α, can improve metabolism (13, 14). But the effects of anti-inflammatory therapies on glycemic control in patients with T2DM were controversial (15–19). Previous meta-analyses have assessed the effects of anti-IL-1 therapies on T2DM (20, 21). However, the totality of the evidence base of the anti-inflammatory therapies on T2DM has not been systematically assessed. We conducted the meta-analyses to clarify the effects of anti-inflammatory therapies on glycemic control in patients with T2DM.

## 2 Methods

The meta-analyses were performed in accordance with the Preferred Reporting Items for Systematic Reviews and Meta-Analyses (PRISMA) guidelines (22).

### 2.1 Search strategy

We searched randomized controlled trials (RCTs) from PubMed, Embase, Web of Science and Cochrane Library from database inception up to 21 September 2022. Search terms include Medical Subject Headings (MeSH), keywords and free-text terms related to anti-inflammatory therapies, type 2 diabetes mellitus, T2DM, fasting plasma glucose, FPG, glycated haemoglobin, HbA1c, C-reactive protein, CRP, anakinra, canakinumab, diacerein, gevokizumab, LY2189102, tocilizumab, salsalate, salicylate, etanercept, remicade, infliximab, adalimumab, enbrel, and dapansutrile. The detailed search strategy is available in **Table S1**. Following the search and removal of duplicates, D Li and J Zhong screened titles and abstracts to identify relevant studies.

### 2.2 Study selection

Studies were eligible for inclusion if they met the following criteria (1): Participants: patients with T2DM; (2) Interventions: at least one of the following treatments was used, anakinra, canakinumab, diacerein, gevokizumab, LY2189102, tocilizumab, salsalate, salicylate, etanercept, remicade, infliximab, adalimumab, enbrel, or dapansutrile; (3) Controls: placebo with or without approved antidiabetic medications, such as metformin, sulfonylureas, and insulin etc.; (4) Outcomes: at least one of the following outcomes was reported, FPG, HbA1c, or CRP; (5) Studies: RCTs. Trials without accessible data or full text were excluded.

### 2.3 Data extraction

Data extraction and analyses from included studies were performed by two authors independently, and conflicts were resolved by a third author. The following information was extracted: first author, publication year, agent, dosage and frequency, follow-up duration, number of participants, patient baseline information (mean age, sex distribution, diabetes duration, baseline BMI, and HbA1c) and outcomes of interest (follow-up FPG, HbA1c, and CRP).

### 2.4 Risk of bias assessment

Risk of bias assessment of the included RCTs was carried out by two authors (D Li and Q Zhang) independently according to the Cochrane Collaboration’s Risk of Bias Tool, which including random sequence generation, allocation concealment, blinding of participants and personnel, blinding of outcome assessment, incomplete outcome data, selective reporting, and other sources of bias.

### 2.5 Data analyses

Continuous variables were expressed as mean difference (MD) with 95% confidence interval (CI). When mean and SD were not available, we calculated from SEM, sample size, median, range, or interquartile range (IQR) using methodology from the Cochrane Library Handbook or the article written by Wan et al. (23, 24). Several studies had more than one intervention groups with different dosages, and for these studies, we chose only one comparable dosage as motioned in **Table 1**. Statistical heterogeneity among studies was assessed with the *I* ^2^ statistic, considering the *I* ^2^ value of 50 - 75% was moderate heterogeneity and above 75% was high heterogeneity (25). We performed subgroup analyses based on the targets of interventions, names of the medication, diabetes duration, follow-up duration, and drug administration regimen. Leave-one-out studies were performed for sensitivity analyses to examine the effect of each trial on the overall analyses. Funnel plot and Egger’s test were used to assess the publication bias and tested for statistical significance. All statistical analyses were performed using Review manager 5.3 and Stata 12.0. A value of p ≤ 0.05 was considered statistically significant.

**Table 1:** Baseline characteristics of included studies ^a^.

First author, year	Agent	Target and mechanism of action	Dosage, frequency	Study duration	Patients randomized, n	Age, years	Male sex, %	Duration of diabetes, year	Baseline BMI	Baseline HbA1c, %
Cardoso CRL 2017^26^	diacerein	TNF-α antagonism in combination with IL-1βR blockade	100 mg/day	48 weeks	84	63.7	20	9	31.3	8.2
Cavelti-Weder C 2012^27^	gevokizumab	IL-1β-specific antibodies	a single dose of 0.03 or 0.1 mg/kg	3 months	48	50	82	9.7	31	9.1
Choudhury RP 2016^17^	canakinumab	IL-1β-specific antibodies	150 mg monthly	12 months	189	61.9	80	–	30.3	6.85
Dominguez H 2005^19^	etanercept	TNF-α inhibition	25 mg twice weekly	4 weeks	19	55	55.6	–	32	7.6
Everett BM 2018^28^	canakinumab	IL-1β-specific antibodies	150 mg once every 3 months	48 months	2303	61	77	–	29.1	7.1
Faghihimani E 2013^29^	salsalate	NF-κB inhibition	3 g/day	12 weeks	60	50.8	–	within 2 months^b^	29.2	5.9
Goldfine AB 2010^30^	salsalate	NF-κB inhibition	3 g/day	14 weeks	54	55.9	55.6	5.1	34	7.8
Goldfine AB 2013^31^	salsalate	NF-κB inhibition	3.5 g/day	48 weeks	286	55.8	52.9	4.9	33.2	7.7
Jangsiripornpakorn J 2022^16^	diacerein	TNF-α antagonism in combination with IL-1βR blockade	50 mg/day	12 weeks	35	52	47.1	11.4	29.5	8.5
Larsen CM 2007^18^	anakinra	IL-1 receptor antagonism	100 mg/day	13 weeks	69	60.3	77.1	11	31.8	8.2
Noe A 2014^32^	canakinumab	IL-1β-specific antibodies	a single dose of 10 mg/kg	24 weeks	86	57.5	68.9	5.1	30.8	7.8
Piovesan F 2017^33^	diacerein	TNF-α antagonism in combination with IL-1βR blockade	50 mg twice daily	90 days	72	62.5	23	13.4	–	8.9
Ramos-Zavala MG 2011^15^	diacerein	TNF-α antagonism in combination with IL-1βR blockade	50 mg once or twice daily	2 months	40	47.8	40	within 6 months^c^	30.8	7.9
Ridker PM 2012^34^	canakinumab	IL-1β-specific antibodies	150 mg/month	4 months	271	54.3	59	4	29.3	7.4
Sloan-Lancaster J 2013^35^	LY2189102	IL-1β-specific antibodies	180 mg/week	24 weeks	42	52.9	25.9	8	32.5	7.9
Tres GS 2018^36^	diacerein	TNF-α antagonism in combination with IL-1βR blockade	50 mg twice daily	12 weeks	71	59	75	14.8	31.3	8.6

Values are given as mean or where not available median.

BMI, body mass index (kg/m^2^); HbA1c, glycated haemoglobin; T2DM, type 2 diabetes mellitus.

a Baseline values are presented for the placebo group.

b Patients were diagnosed with T2DM within 2 months.

c Patients were diagnosed with T2DM within 6 months.

## 3 Results

### 3.1 Included studies and baseline characteristics

**Figure 1:**
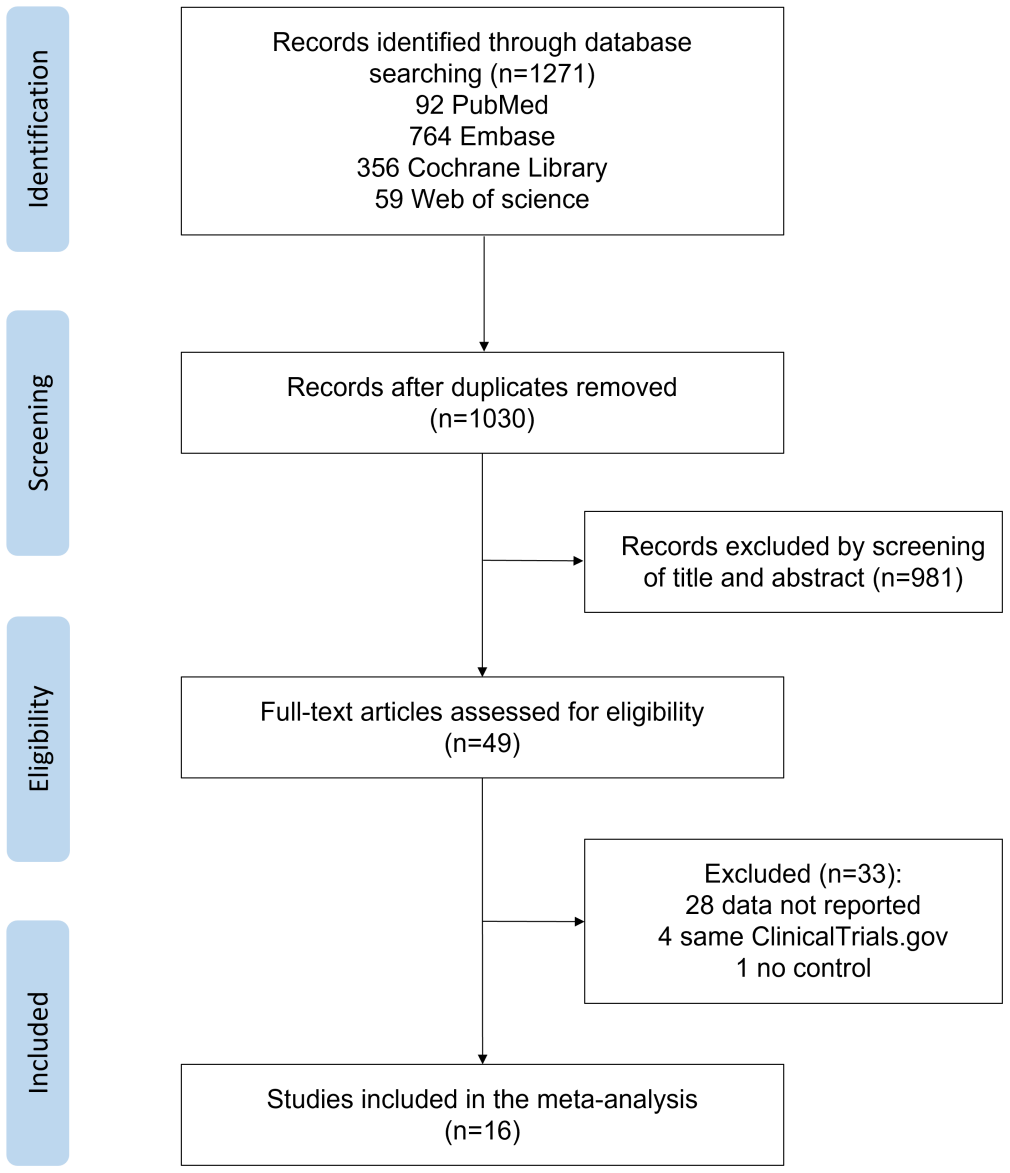
Flowchart diagram of study selection process.

**Figure 1** shows the details of the literature search and selection process. Of 1271 reports identified, 241 reports were excluded due to duplication, and 981 were excluded based on titles and abstracts. Of 49 reports reviewed in full, 33 were excluded based on eligibility criteria. A total of 16 reports involving 3729 participants with T2DM were included in the final analyses (15–19, 26–36). **Table 1** shows the baseline characteristics of the 16 RCTs. Trials included were published between 2005 and 2022. The follow-up duration was between 1 and 48 months. Trails reported by Everett et al. (28) had the longest follow-up duration (48 months), which was not comparable with all the others, and a more comparable time point (6 months) was used in the subsequent analyses. Among the 16 trails, 4 trails were for canakinumab (17, 28, 32, 34), 5 trails for diacerein (15, 16, 26, 33, 36), 3 trails for salsalate (29–31), and the rest were for anakinra (18), gevokizumab (27), LY2189102 (35), and etanercept (19). The dosage and frequency of the treatments are shown in **Table 1**.

### 3.2 Risk of bias of individual studies

The quality of the included trials was assessed according to the criteria of the Cochrane Handbook. A detailed evaluation of the risk of bias for each clinical trial and risk of bias summary are presented in **Figure S1**. Among the 16 RCTs, only 1 was judged to be at high risk of bias as an open-label randomized trial (19), 6 were judged to be at low risk of bias and 9 as being at unclear risk of bias. Unclear risks were related to selection bias, reporting bias, and other bias.

### 3.3 Meta-analyses

#### 3.3.1 FPG

**Figure 2:**
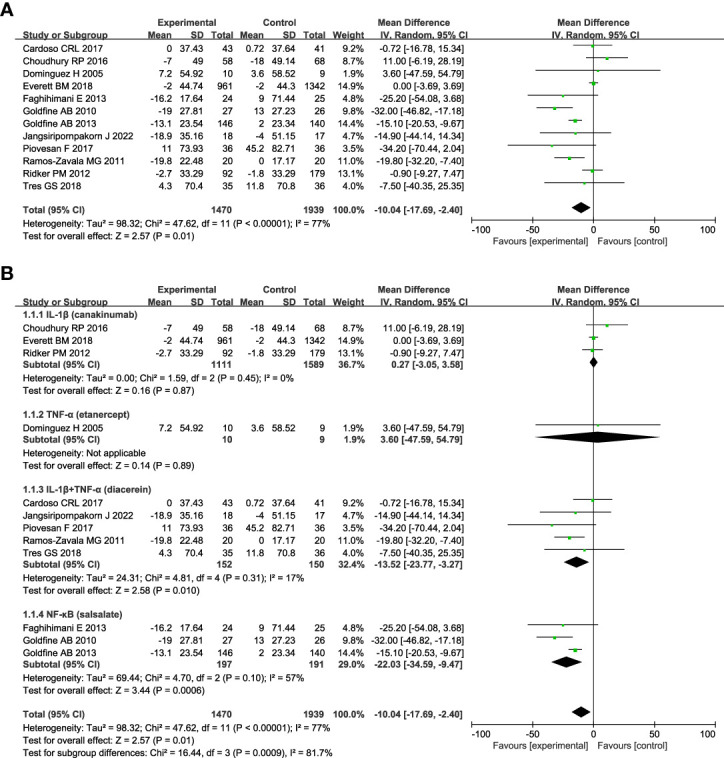
Forest plot of pooled mean difference in change in FPG (mg/dL). **(A)** Meta-analyses of the effects of anti-inflammatory therapies on FPG in patients with T2DM; **(B)** The forest plot of FPG in subgroup analyses defined by the targets of interventions. fasting plasma glucose, FPG; CI, confidence interval; IV, inverse variance; SD, standard deviation.

**Figure 2** shows anti-inflammatory therapies can significantly decrease the level of FPG (n = 12; MD = - 10.04; 95% CI: - 17.69, - 2.40; P =0.01) compared with control, and there was statistically significant heterogeneity among studies (*I^2^* = 77%; P < 0.00001) (**Figure 2A**). We did a series of subgroup analyses of FPG based on the targets of interventions, diabetes duration, and follow-up duration. Subgroup analyses based on the targets of interventions show that drugs targeting IL-1β plus TNF-α (diacerein) (n = 5; MD = - 13.52; 95% CI: - 23.77, - 3.27; P =0.01) or NF-κB alone (salsalate) (n = 3; MD = - 22.03; 95% CI: - 34.59, - 9.47; P =0.0006) can significantly decrease the level of FPG compared with control, whereas drugs targeting IL-1β (canakinumab) or TNF-α (etanercept) alone had no significant effect on the change of FPG (**Figure 2B**). Patients with T2DM less than 3 years since diagnosis (n=2, MD = - 20.64; 95% CI: - 32.03, - 9.25; P =0.0004) seem to benefit more from anti-inflammatory therapies than those between 3 and 10 years (n=3, MD = - 14.79; 95% CI: - 28.69, - 0.89; P =0.04), and no significant effect was found in those more than 10 years (n=4, MD = - 7.94; 95% CI: - 20.17, 4.3; P =0.2) (**Figure S2A**). Anti-inflammatory therapies can decrease the level of FPG in patients whose follow-up duration was less than or equal to 3 months (n=6, MD = - 19.01; 95% CI: - 28.57, -9.45; P < 0.0001), but no significant effect was found in patients with longer follow-up duration (**Figure S2B**).

#### 3.3.2 HbA1c

**Figure 3:**
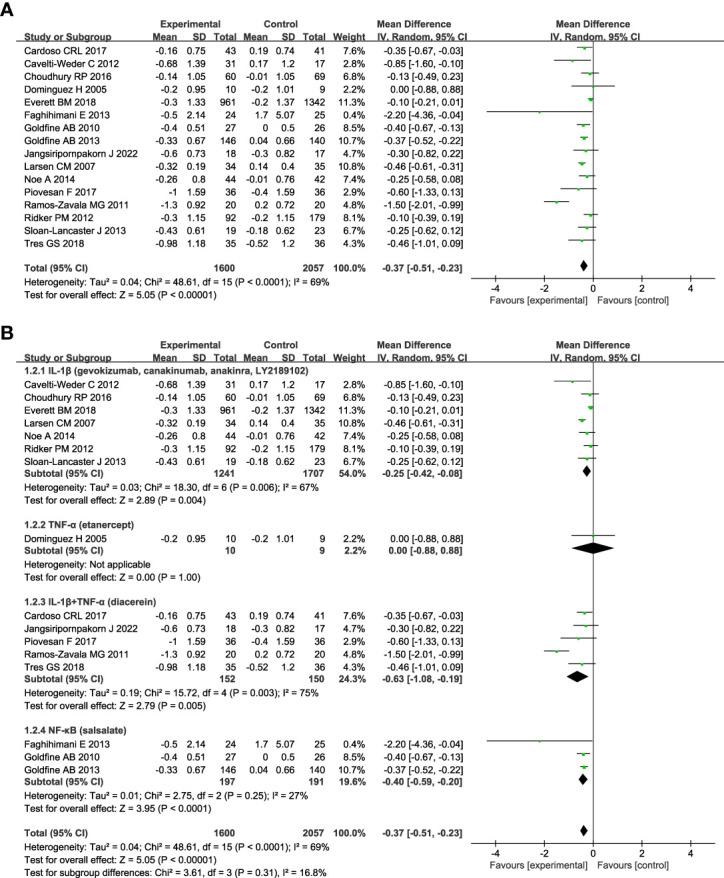
Forest plot of pooled mean difference in change in HbA1c (%). **(A)** Meta-analyses of the effects of anti-inflammatory therapies on HbA1c in patients with T2DM; **(B)** The forest plot of HbA1c in subgroup analyses defined by the targets of interventions. glycated haemoglobin, HbA1c; CI, confidence interval; IV, inverse variance; SD, standard deviation.

The change in HbA1c was assessed in all studies. **Figure 3A** shows anti-inflammatory therapies can significantly decrease the level of HbA1c (n = 16; MD = - 0.37; 95% CI: - 0.51, - 0.23; P < 0.00001) with moderate heterogeneity among studies (*I* ^2^ = 69%; P < 0.0001) (**Figure 3A**). The sensitivity analyses of HbA1c indicated the stability of the results (**Figure S3**). Subgroup analyses based on the targets of the interventions show that drugs targeting IL-1β plus TNF-α (diacerein) (n=5; MD = - 0.63; 95% CI: - 1.08, - 0.19; P =0.005) can reduce the level of HbA1c better than targeting IL-1β (gevokizumab, canakinumab, anakinra, or LY2189102) (n=7; MD = - 0.25; 95% CI: - 0.42, - 0.08; P = 0.004) or TNF-α (etanercept) (n=1; MD = 0.00; 95% CI: - 0.88, 0.88; P =1.00) alone (**Figure 3B**). Anti-inflammatory therapies targeting NF-κB (salsalate) (n = 3; MD = - 0.40; 95% CI: - 0.59, - 0.20; P < 0.0001) can significantly decrease the level of HbA1c compared with control, and there was no heterogeneity among studies (*I* ^2^ = 27%; P = 0.25). Subgroup analyses according to the name of the medications show in **Figure S4A**, gevokizumab (n = 1; MD = - 0.85; 95% CI: - 1.60, - 0.10; P =0.03) can reduce the level of HbA1c more than diacerein (n = 5; MD = - 0.63; 95% CI: - 1.08, - 0.19; P =0.005), anakinra (n =1; MD = - 0.46; 95% CI: - 0.61, - 0.31; P < 0.00001), salsalate (n = 3; MD = - 0.40; 95% CI: - 0.59, - 0.20; P < 0.0001), and canakinumab (n = 4; MD = - 0.11; 95% CI: - 0.21, - 0.02; P = 0.02). LY2189102 and etanercept had no significant effect on HbA1c compared with the control. Subgroup analyses based on diabetes duration show that more reduction of HbA1c was seen in patients with T2DM less than 3 years since diagnosis (n = 2, MD = -1.54; 95% CI: - 2.04, - 1.04; P < 0.00001) than those between 3 and 10 years (n = 6, MD = - 0.32; 95% CI: - 0.43, - 0.21; P < 0.00001), and those more han 10 years (n = 5, MD = - 0.44; 95% CI: - 0.56, - 0.31; P < 0.00001) (**Figure S4B**). Anti-inflammatory therapies were more effective in patients whose follow-up duration was less than or equal to 3 months (n = 7, MD = - 0.71; 95% CI: - 1.16, - 0.26; P = 0.002) (**Figure S4C**). Repeated drug administration regimen (n = 14, MD = - 0.37; 95% CI: - 0.52, -0.21; P < 0.00001) and single dosing (n = 2, MD = - 0.45; 95% CI: - 1.01, 0.10; P = 0.11) had similar effects on HbA1c (**Figure S4D**).

#### 3.3.3 CRP

**Figure 4:**
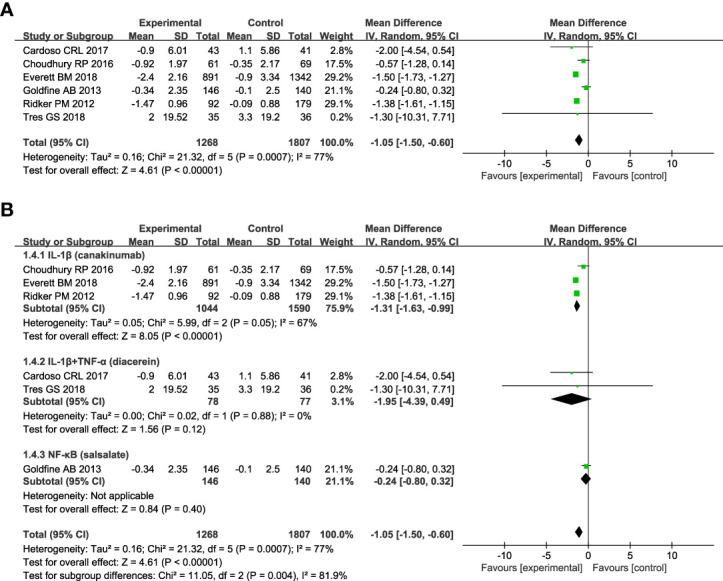
Forest plot of pooled mean difference in change in CRP (mg/L). **(A)** Meta-analyses of the effects of anti-inflammatory therapies on CRP in patients with T2DM; **(B)** The forest plot of CRP in subgroup analyses defined by the targets of interventions. C-reactive protein, CRP; CI, confidence interval; IV, inverse variance; SD, standard deviation.

**Figure 4** shows anti-inflammatory therapies can decrease the level of CRP compared with control (n = 6; MD = - 1.05; 95% CI: - 1.50, - 0.60; P < 0.00001), and there was high heterogeneity among studies (*I* ^2^ = 77%; P = 0.0007) (**Figure 4A**). Subgroup analyses based on the targets of interventions show that drugs targeting IL-1β (canakinumab) can significantly reduce the level of CRP (n = 3; MD = - 1.31; 95% CI: - 1.63, - 0.99; P < 0.00001), whereas no significant effect was found in drugs targeting IL-1β plus TNF-α (diacerein) (n = 2; MD = - 1.95; 95% CI: - 4.39, 0.49; P =0.12) or NF-κB (salsalate) (n = 1; MD = - 0.24; 95% CI: - 0.80, 0.32; P =0.40) (**Figure 4B**).

#### 3.3.4 Publication bias

Egger’s test for HbA1c suggested significant publication bias (p = 0.003) (**Figure S5**). However, the effect was the same as the original effect after using Duval and Tweedie’s trim and fill, and the result showed that no trimming was performed, and the data stayed unchanged.

## 4 Discussion

Our meta-analyses of 16 RCTs published between 2005 and 2022 examined the effects of anti-inflammatory therapies on glycemic control in patients with T2DM. Two previous meta-analyses published in 2018 and 2019, concluded that anti-IL-1 therapies can significantly decrease the level of HbA1c and CRP, and have mild hypoglycaemic effect on patients with T2DM (20, 21). However, the effects of anti-inflammatory therapies targeting other inflammatory molecules and the overall effects of anti-inflammatory therapies on T2DM remain to be discovered. Therefore, we performed further analyses of anti-inflammatory therapies based on different inflammatory targets, including IL-1β, IL-1βR, TNF-α, and NF-κB. Our results show that anti-inflammatory therapies, including anti-IL-1 therapies, can significantly decrease the level of FPG, HbA1c and CRP in patients with T2DM. Our findings indicate the clinical efficacy of treating T2DM based on the pathogenesis of diabetes and give suggestions for the future anti-inflammatory clinical trials.

Chronic low-grade inflammation was found in diabetic islets, with increased innate immune cell infiltration and cytokine secretion (37). Immune cell infiltration and cytokine release directly impairs β cell mass and function (38). IL-1β was the first described proinflammatory cytokine in the islets of patients with T2DM (39). IL-1β impairs β cell function and induces the apoptosis of β cells (40). Block IL-1β signaling pathway by antagonists or antibodies had beneficial effects on β cell function and glycemic control in patients with T2DM (41, 42). Anakinra, a recombinant human IL-1βR antagonist, can significantly reduce the level of HbA1c and may improve glycemic control by increasing insulin secretion (18). Canakinumab, gevokizumab and LY2189102 are recombinant human engineered monoclonal antibodies, which can neutralize the activity of IL-1β by forming a complex with circulating IL-1β. Canakinumab can also reduce the blood levels of IL-6 and CRP (17). All the anti-IL-1β therapies mentioned above had significant effect on glucose control as reflected by reductions in HbA1c, which was also reported by previous meta-analyses (20, 21). However, some of the beneficial effects were only detected by certain treatment periods, not the whole follow-up periods (28, 35). As shown in our subgroup analyses, anti-inflammatory therapies may work better in patients with short follow-up duration (less than or equal to 3 months). LY2189102 can improve blood glucose control for 12 weeks, but the effect was attenuated over time and there was no difference at 24 weeks (35). The study reported by Everett BM et al. showed that canakinumab can reduce HbA1c during the first 6 to 9 months of treatment, but no significant effect was found by the end of the follow-up period at 48 months (28). The exact reason for this attenuation is unclear, but the availability of other antidiabetic therapies and lifestyle interventions may contribute to this phenomenon (28).

TNF-α can diminish glucose-dependent insulin secretion and impair the function of β cells both *in vitro* and *in vivo* (43, 44). But etanercept, a TNF-α inhibitor, has no significant effect on FPG or HbA1c (19). Etanercept can improve the glucose tolerance of some individuals, but no significant effect was found in the whole group (19). It was difficult to say whether etanercept has a positive effect on β cells since no more than 20 individuals was included in this clinical trial, and studies with a larger number of patients with T2DM are needed to elucidate this issue.

Diacerein is both an IL-1βR blocker and a TNF antagonist. It can inhibit the synthesis and activity of IL-1 and TNF-α by its active metabolite rhein (45). Diacerein can reduce the HbA1c level without affecting the homeostasis model assessment-insulin resistance (HOMA-IR), indicating that it may play a role in insulin secretion (36). And a higher dosage of diacerein (100 mg/day) may be more effective in improving the glycemic outcome (16). Our results show that interventions targeting IL-1β plus TNF-α can reduce the level of HbA1c better than targeting IL-1β or TNFα alone in patients with T2DM. Diacerein had no significant effect on CRP in patients with T2DM, though reduced TNF-α was observed (26, 36). Those studies were carried out in patients with longer duration of diabetes, and most participants were undergoing treatment with metformin, statins, sulfonylureas, or renin-angiotensin system blockers, which have potential roles in anti-inflammation, and might attenuate the anti-inflammatory effect of diacerein (13, 26, 36).

Salsalate, a prodrug form of salicylate, shows anti-inflammatory effects by inhibiting the IKKb/NF-κB and JNK signaling pathways (46, 47). Salsalate can improve glycemic control by affecting cellular kinases nonspecifically and increasing insulin secretion of β cells (48). After 1 year treatment, salsalate still had effects on HbA1c and FPG in patients with T2DM (31). Salsalate can decrease the level of inflammatory mediators, such as leukocytes, neutrophils, and lymphocytes, but had little effect on CRP in patients with T2DM (31). T2DM seems to result from a long-term process of inflammation, even years before diagnosis (35). Greater benefits of salsalate might be seen in patients with newly diagnosed T2DM or longer treatment duration.

Our results show that patients with newly diagnosed T2DM may benefit more from anti-inflammatory therapies. However, Kataria Y et al. reported that the effects of anti-IL-1β therapies depend on the baseline dysmetabolic status, and patients with a more metabolic imbalance at baseline may benefit more after treatment (21). The differences between our studies may come from the different types of medications analyzed, as we included lots of anti-inflammatory medications, not just IL-1β antibodies and IL-1βR antagonists. Since no newly diagnosed T2DM patients were included in the studies of anti-IL-1β therapies, the effects of anti-IL-1β therapies on those patients remain to be seen.

There are some limitations in our study. First, lifestyle modification and antidiabetic medications were allowed in most of the included trials, which may affect or attenuate the efficacy of anti-inflammatory therapies. Second, most of the follow-up duration varied from 1 to 12 months, and longer clinical trials are needed since medication efficacy may change over time. Finally, publication bias exists in the meta-analyses, but the results stay the same after a trim and fill analysis.

## 5 Conclusions

This study helps us better understand the possibility and efficiency of anti-inflammatory therapies for T2DM based on the pathogenetic processes of the disease. The present analyses demonstrated that targeting cytokines, cytokine receptors, and inflammation-associated nuclear transcription factors, such as IL-1β, IL-1βR, TNF-α, and NF-κB, alone or in combination can significantly reduce the level of FPG, HbA1c, and CRP in patients with T2DM. In addition, patients with a short duration of T2DM may benefit more from anti-inflammatory therapies. Since anti-inflammatory medications can reduce inflammation throughout the body, these medications may be used to treat diseases with similar pathologies, such as cardiovascular disease, chronic kidney disease, and rheumatic arthritis with or without T2DM.

## Data availability statement

The original contributions presented in the study are included in the article/**Supplementary Material**. Further inquiries can be directed to the corresponding author.

## Author contributions

DL and JinZ conceived and designed the study. DL and JiaZ did the scientific literature search and data extraction of the included studies. DL and QZ did the quality assessment and carried out the analyses. DL wrote the first draft of the present manuscript. All authors contributed to the article and approved the submitted version.
