# Supplementary material for: Effects of anti-inflammatory therapies on glycemic control in type 2 diabetes mellitus
Source: Front Immunol. 2023 Mar 1;14:1125116. doi: 10.3389/fimmu.2023.1125116 (PMC10014557; doi:10.3389/fimmu.2023.1125116)
Supplement: Supplementary file 1 [file DataSheet_1.docx]

Supplementary Material

**Effects of Anti-inflammatory Therapies on Glycemic Control in Type 2 Diabetes Mellitus**

# Supplementary Figures and Tables

## Supplementary Figures

**Supplementary Figure 1.** Risk of bias and risk of bias summary. (A) Risk of bias graph. (B) Risk of bias summary. In Figure S1A, green represents a low risk of bias, yellow represents unclear risk of bias, while red represents high risk of bias. In Figure S1B, + represents a low risk of bias, - represents a high risk of bias, while ? represents unclear risk of bias.

**Supplementary Figure 2.** Subgroup analyses of pooled mean difference in change in FPG (mg/dL). The forest plot of FPG in subgroup analyses defined by the diabetes duration (A), and follow-up duration (B). Abbreviations: fasting plasma glucose, FPG; CI, confidence interval; IV, inverse variance; SD, standard deviation.

**Supplementary Figure 3.** Sensitivity analyses in HbA1c of studies included. Abbreviations: glycated haemoglobin, HbA1c; CI, confidence interval.

**Supplementary Figure 4.** Subgroup analyses of pooled mean difference in change in HbA1c (%). The forest plot of HbA1c in subgroup analyses defined by the name of intervention (A), diabetes duration (B), follow-up duration (C), and the drug administration regimen (D). Abbreviations: glycated haemoglobin, HbA1c; CI, confidence interval; IV, inverse variance; SD, standard deviation.

**Supplementary Figure 5.** Funnel plot for publication bias test of included studies for HbA1c. Abbreviations: glycated haemoglobin, HbA1c; standard mean difference, SMD.

## Supplementary Tables

**Supplementary Table 1.** Full search strategy in database.

A


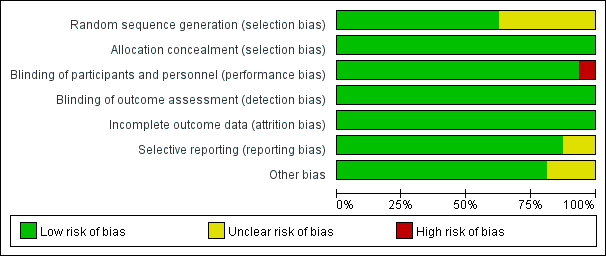


B


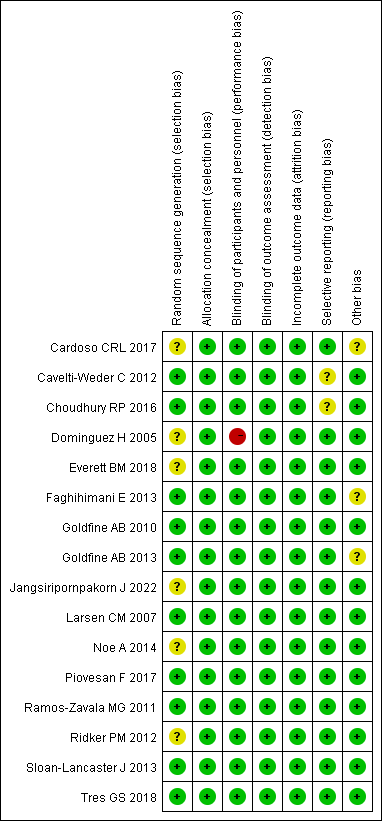


**Supplementary Figure 1.** Risk of bias and risk of bias summary. (A) Risk of bias graph. (B) Risk of bias summary. In Figure S1A, green represents a low risk of bias, yellow represents unclear risk of bias, while red represents high risk of bias. In Figure S1B, + represents a low risk of bias, - represents a high risk of bias, while ? represents unclear risk of bias.

A


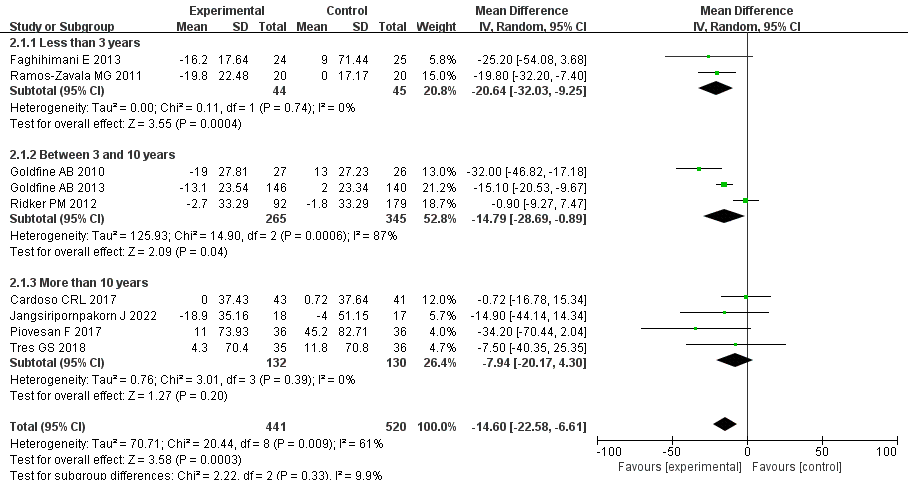


B


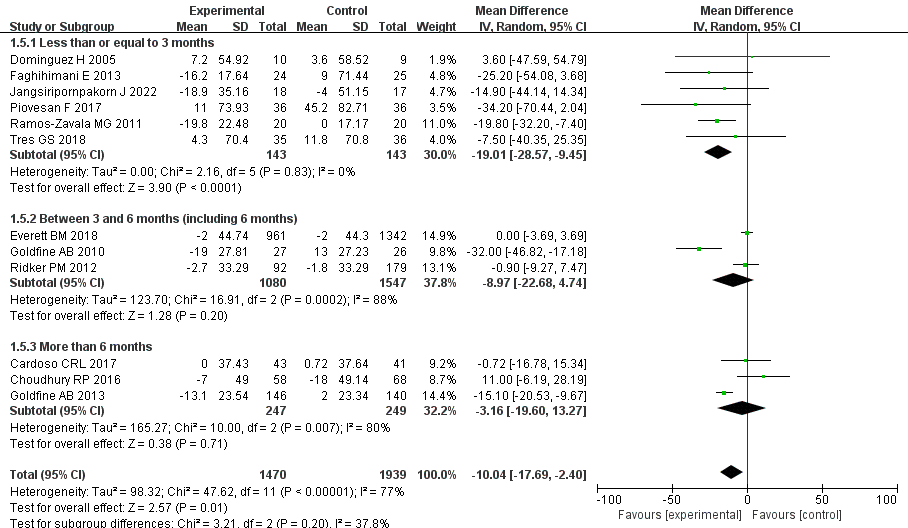


**Supplementary Figure 2.** Subgroup analyses of pooled mean difference in change in FPG (mg/dL). The forest plot of FPG in subgroup analyses defined by the diabetes duration (A), and follow-up duration (B). Abbreviations: fasting plasma glucose, FPG; CI, confidence interval; IV, inverse variance; SD, standard deviation.

**Supplementary Figure 3.** Sensitivity analyses in HbA1c of studies included. Abbreviations: glycated haemoglobin, HbA1c; CI, confidence interval.

A


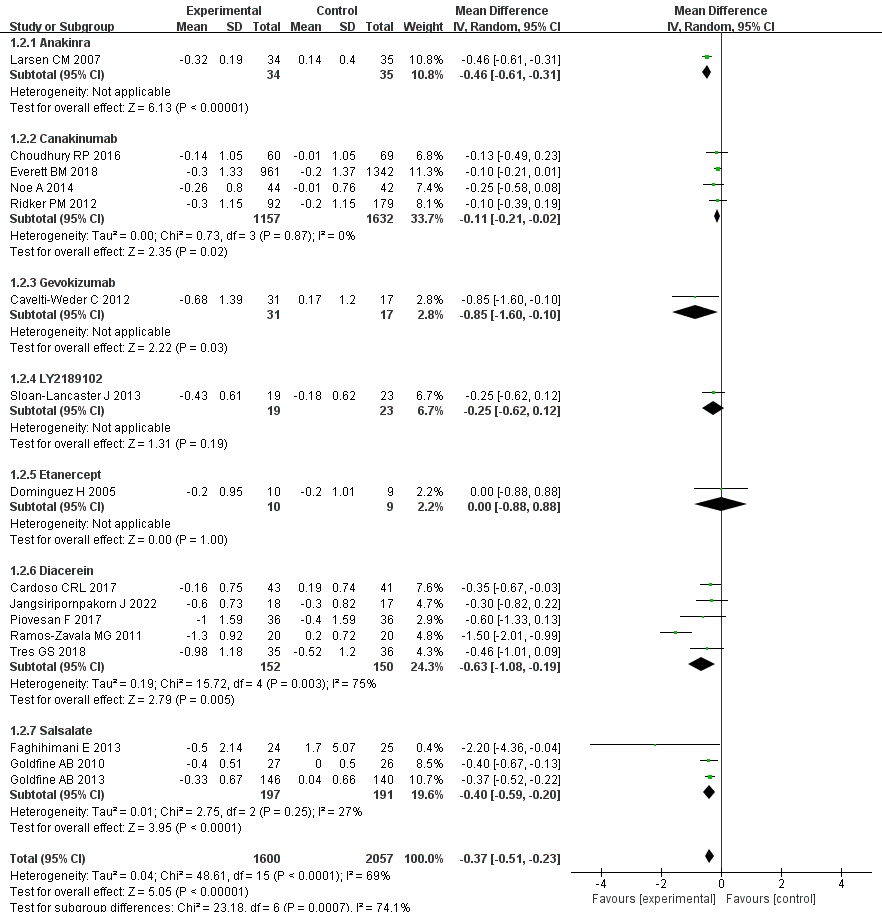


B


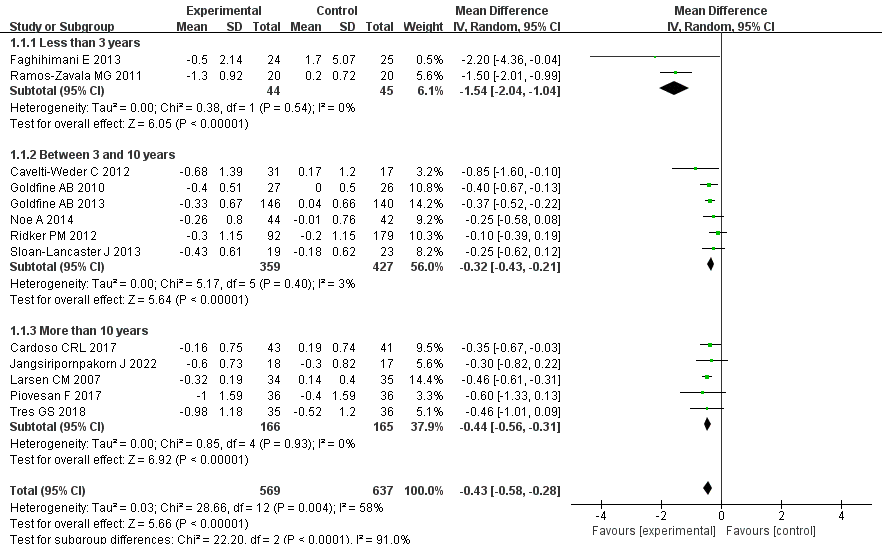


C


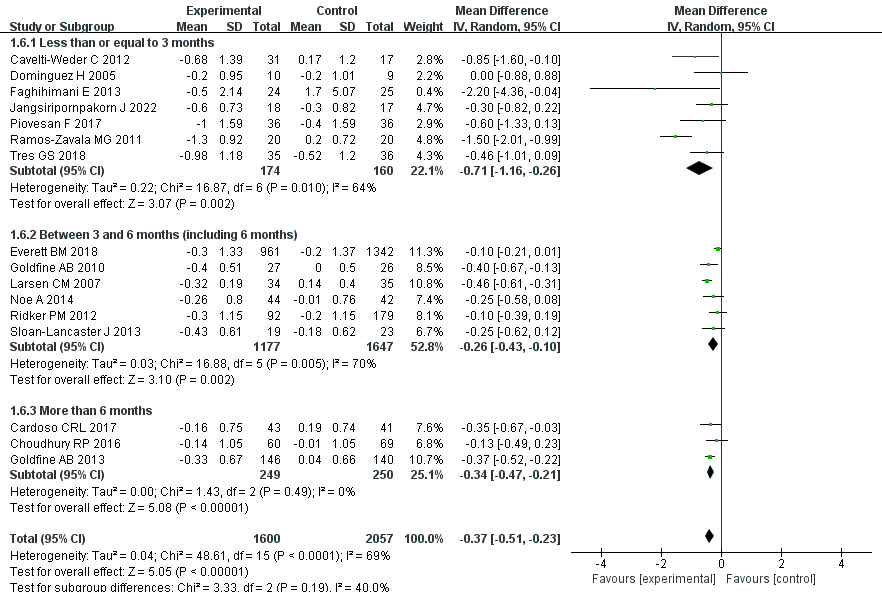


D


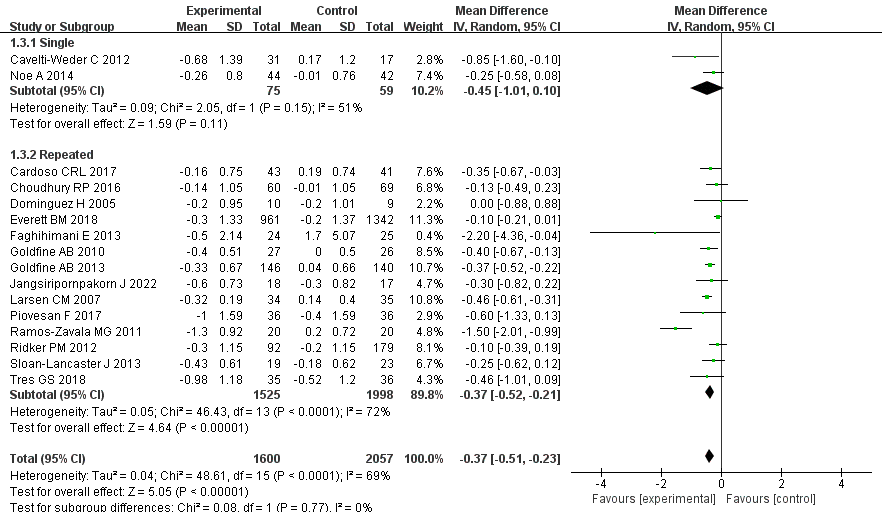


**Supplementary Figure 4.** Subgroup analyses of pooled mean difference in change in HbA1c (%). The forest plot of HbA1c in subgroup analyses defined by the name of intervention (A), diabetes duration (B), follow-up duration (C), and the drug administration regimen (D). Abbreviations: glycated haemoglobin, HbA1c; CI, confidence interval; IV, inverse variance; SD, standard deviation.

**Supplementary Figure 5.** Funnel plot for publication bias test of included studies for HbA1c. Abbreviations: glycated haemoglobin, HbA1c; standard mean difference, SMD.

**Supplementary Table 1.** Full search strategy in database.

| PubMed |
| --- |
| Search date: 21 September 2022 |
| Search results: 92 |
| #1 "Anti-Inflammatory Agents"[MeSH Terms]  #2 "anti-inflammatory"[All Fields] OR "Anakinra"[All Fields] OR "Canakinumab"[All Fields] OR "Diacerein"[All Fields] OR "Gevokizumab"[All Fields] OR "LY2189102"[All Fields] OR "Tocilizumab"[All Fields] OR "Salsalate"[All Fields] OR "Salicylate"[All Fields] OR "Etanercept"[All Fields] OR "Remicade"[All Fields] OR "Infliximab"[All Fields] OR "Adalimumab"[All Fields] OR "Enbrel"[All Fields] OR "Dapansutrile"[All Fields]  #3 #1 OR #2  #4 "diabetes mellitus, type 2"[MeSH Terms]  #5 "diabetes mellitus, type 2"[All Fields] OR "Type 2 diabetes"[All Fields] OR "T2D"[All Fields] OR "T2DM"[All Fields] OR " non-insulin-dependent diabetes mellitus "[All Fields] OR "NIDDM"[All Fields]  #6 #4 OR #5  #7 "glucose"[All Fields] OR "fasting plasma glucose"[All Fields] OR "FPG"[All Fields] OR "glycated haemoglobin"[All Fields] OR "HbA1c"[All Fields] OR "C-reactive protein"[All Fields] OR "CRP"[All Fields]  #8 "Randomized Controlled Trial"[Publication Type] OR "Randomized Controlled Trial"[All Fields] OR "Trial*"[All Fields]  #9 #3 AND #6 AND #7 AND #8 |
| Embase |
| Search date: 21 September 2022 |
| Search results: 764 |
| #1 'Anti-Inflammatory Agents'/exp  #2 anti-inflammatory OR Anakinra OR Canakinumab OR Diacerein OR Gevokizumab OR LY2189102 OR Tocilizumab OR Salsalate OR Salicylate OR Etanercept OR Remicade OR Infliximab OR Adalimumab OR Enbrel OR Dapansutrile  #3 #1 OR #2  #4 'non insulin dependent diabetes mellitus'/exp  #5 'diabetes mellitus, type 2' OR 'type 2 diabetes' OR T2D OR T2DM OR NIDDM  #6 #4 OR #5  #7 glucose OR 'fasting plasma glucose' OR FPG OR 'glycated haemoglobin' OR HbA1c OR 'C-reactive protein' OR CRP  #8 'Randomized Controlled Trial' OR trial*  #9 #3 AND #6 AND #7 AND #8  Cochrane Library  Search date: 21 September 2022  Search results: 356  #1 MeSH descriptor: [Anti-Inflammatory Agents] explode all trees  #2 (anti-inflammatory) OR (Anakinra) OR (Canakinumab) OR (Diacerein) OR (Gevokizumab) OR (LY2189102) OR (Tocilizumab) OR (Salsalate) OR (Salicylate) OR (Dapansutrile) OR (Etanercept) OR (Remicade) OR (Infliximab) OR (Adalimumab) OR (Enbrel)  #3 #1 OR #2  #4 MeSH descriptor: [Diabetes Mellitus, Type 2] explode all trees  #5 (Diabetes Mellitus, Type 2) OR (Type 2 diabetes) OR (non-insulin-dependent diabetes mellitus) OR (T2D) OR (T2DM) OR (NIDDM)  #6 #4 OR #5  #7 (Glucose) OR (fasting plasma glucose) OR (FPG) OR (Glycated haemoglobin) OR (HbA1c) OR (C-reactive protein) OR (CRP)  #8 #3 AND #6 AND #7  Web of Science  Search date: 21 September 2022  Search results: 59  #1 TI=([anti-inflammatory OR Anakinra OR Canakinumab OR Diacerein OR Gevokizumab OR LY2189102 OR Tocilizumab OR Salsalate OR Salicylate OR Etanercept OR Remicade OR Infliximab OR Adalimumab OR Enbrel OR Dapansutrile) OR AB=([anti-inflammatory OR Anakinra OR Canakinumab OR Diacerein OR Gevokizumab OR LY2189102 OR Tocilizumab OR Salsalate OR Salicylate OR Etanercept OR Remicade OR Infliximab OR Adalimumab OR Enbrel OR Dapansutrile) OR AK=([anti-inflammatory OR Anakinra OR Canakinumab OR Diacerein OR Gevokizumab OR LY2189102 OR Tocilizumab OR Salsalate OR Salicylate OR Etanercept OR Remicade OR Infliximab OR Adalimumab OR Enbrel OR Dapansutrile)  #2 TI=("diabetes mellitus, type 2" OR "Type 2 diabetes" OR T2D OR T2DM OR "non-insulin-dependent diabetes mellitus" OR NIDDM) OR AB=("diabetes mellitus, type 2" OR "Type 2 diabetes" OR T2D OR T2DM OR "non-insulin-dependent diabetes mellitus" OR NIDDM) OR AK=("diabetes mellitus, type 2" OR "Type 2 diabetes" OR T2D OR T2DM OR "non-insulin-dependent diabetes mellitus" OR NIDDM)  #3 TI=(glucose OR "fasting plasma glucose" OR FPG OR "Glycated haemoglobin" OR HbA1c OR "C-reactive protein" OR CRP) OR AB=( glucose OR "fasting plasma glucose" OR FPG OR "Glycated haemoglobin" OR HbA1c OR "C-reactive protein" OR CRP) OR AK=( glucose OR "fasting plasma glucose" OR FPG OR "Glycated haemoglobin" OR HbA1c OR "C-reactive protein" OR CRP)  #4 TI=(“Randomized Controlled Trial” OR trial*) OR AB=(“Randomized Controlled Trial” OR trial*) OR AK=(“Randomized Controlled Trial” OR trial*)  #5 #1 AND #2 AND #3 AND #4 |
